# Acute smartphone use impairs vigilance and inhibition capacities

**DOI:** 10.1038/s41598-023-50354-3

**Published:** 2023-12-27

**Authors:** Thomas Jacquet, Romuald Lepers, Benjamin Pageaux, Bénédicte Poulin-Charronnat

**Affiliations:** 1 CAPS, Inserm U1093, Université de Bourgogne, Faculté Des Sciences du Sport, BP 27877 UFR STAPS, I3M, 64 Rue de Sully, 21000 Dijon, France; 2 École de kinésiologie et des Sciences de l’activité physique (EKSAP), Faculté de médecine, Université de Montréal, Montreal, QC Canada; 3 Centre de recherche de l’Institut universitaire de geriatrie de Montréal (CRIUGM), Montreal, QC Canada; 4 Centre interdisciplinaire de recherche sur le cerveau et l’apprentissage (CIRCA), Montreal, QC Canada; 5 LEAD – CNRS UMR5022, Université de Bourgogne, Pôle AAFE, 21000 Dijon, France

**Keywords:** Human behaviour, Cognitive neuroscience

## Abstract

Smartphones are now in very widespread use, and concerns have arisen about potential detrimental effects, even with acute use. These adverse consequences are often linked to the emergence of mental fatigue. While the cognitive implications of fatigue are well-documented, knowledge about the specific influence of acute smartphone use on cognitive performance remains scarce. The aim of this study was therefore to investigate the impact of acute smartphone use on cognitive performance. It included two experiments: one designed to assess the impact of smartphone use on vigilance, and the other focusing on evaluating inhibition capacities. In Experiment 1, two groups of 40 participants completed a Psychomotor Vigilance Task (PVT) before and after using a smartphone for 45 min (experimental group), or before and after watching a documentary (control group). In Experiment 2, two groups of 40 participants were subjected to a similar experimental design but had to perform a Go/NoGo task instead of a PVT. Mental fatigue and drowsiness were evaluated with visual analog scales before and after smartphone use and watching a documentary. Results suggested that both watching a documentary and using a smartphone for 45 min increased subjective mental fatigue and drowsiness. Watching the documentary did not impair cognitive performance. Reaction times on the PVT and number of errors on NoGo trials in the Go/NoGo task were higher among the participants in the smartphone condition. These results indicate reduced vigilance and impaired inhibition capacities only after smartphone use. We conclude that acute smartphone use induces mental fatigue and decreases cognitive performance. Further research is needed to understand the mechanisms underlying this decline in cognitive performance.

## Introduction

The smartphone is playing a growing role in society as a key everyday tool for communication and information sharing. While only 35% of Americans said they owned a smartphone in 2011, that number had increased to about 85% in 2021^1^. According to a survey, users spend more than 20 h per week texting, emailing, and using social media, demonstrating a high level of reliance on smartphones for social interaction and communication^2^. However, even if the smartphone is a valuable tool that improves our daily lives by facilitating communication, task management and our access to entertainment, negative side effects have also been reported.

When used regularly over a period of several months, the smartphone can negatively affect sleep quality^3^ and mood^4^. Furthermore, a positive correlation between smartphone addiction, depression and anxiety levels has been found in adolescents^3^. This both reduces life happiness and increases the likelihood of future health risks^5^. Intensive smartphone use could also exacerbate musculoskeletal disorders due to the high level of muscle activity solicited in the upper body parts, i.e., neck extensors, upper trapezius, and erector spinae^6^. Moreover, extended periods of smartphone use are related to lower physical fitness, including decreased flexibility and strength^7^.

Chronic smartphone use can also impair cognitive performance. Intensive smartphone users have poorer numerical processing capacities^8^ than nonusers and poorer inhibitory control^9^ than “normal” users. Moreover, three months of smartphone use has been found to lead to a decline in performance on an arithmetic task in nonsmartphone users^8^.

Although the impacts of chronic smartphone use (e.g., repeated sessions) on cognitive and physical performances are receiving increasing attention, studies of the effects of acute use (e.g., a single session) remain rare. In 2017, Greco et al.^10^ found evidence that acute smartphone use for 30 min can bring about a decrease in physical and technical performances among young football players. A decline in performance has also been observed in other sports with, for example, an impairment of visuomotor abilities in volleyball players^11^, decision-making in boxers^12^, or an increase in the time taken to cover 50 m by swimmers^13^. However, to the best of our knowledge, only a few studies have investigated the effect of acute smartphone use on cognitive performance^12,14,15^. In these studies, all conducted by the same group of researchers, the acute use of a smartphone increased reaction times and/or errors during a subsequent Stroop task. It is important to note that the authors of studies that have examined the effects of acute smartphone use have also specified the activity to be performed during this period of use. Participants have had to either play games^10^ or use social networks^11–15^. While this approach makes it possible to control the experimental conditions better, it should be noted that it may also be less ecological than free and unrestricted smartphone use. Furthermore, these studies all had low sample sizes (*N* ≤ 20) and replications with a larger sample size are therefore needed in order to confirm or challenge the observed results. All these studies found a significant increase in the subjective feelings of mental fatigue after acute smartphone use. For this reason, the authors attributed the adverse effects of smartphone use on subsequent performance to the induction of mental fatigue.

Mental fatigue is a common phenomenon that is defined as a psychobiological state caused by prolonged periods of demanding cognitive activity. Mental fatigue is characterized by an increase in subjective feelings of “tiredness” and “lack of energy”^16^, associated with a decrease in cognitive performance or an increase in the effort required to maintain performance^17^. It is usually induced by means of controlled laboratory tasks, such as the Stroop task, performed for 30 min or more^18^. The effects of mental fatigue on both cognitive and physical performances are well documented. Mental fatigue impairs sport-related decision-making^19^, technical skills^20^, motor control^21^, and endurance^22^ performances. All the adverse effects lead to a decrease in sporting performances, in particular in the case of soccer^23^, table tennis^24^, cricket^25^, golf or swimming^26^ performances.

Cognitive performances are also impaired by mental fatigue. For instance, Smith et al.^27^ demonstrated that mental fatigue induced by a 45-min AX-CPT or Stroop task resulted in a decreased vigilance, characterized by sustained alertness over time and evidenced by an approximate 4% increase in reaction times during a subsequent psychomotor vigilance task (PVT). In addition to vigilance, attention, which requires a selective focus on specific stimuli or tasks, may also be impaired as a result of mental fatigue^28^. Furthermore, mental fatigue has been found to negatively impact executive functions, including mental flexibility^29^, planning^29^, decision-making^30^, emotional regulation^31^, and inhibition capacities^32,33^. Inhibition capacities have often been evaluated using Go/NoGo tasks, and studies have shown that performance declines over time during prolonged Go/NoGo tasks^32^. Moreover, Guo et al.^33^ demonstrated that performing a mentally fatiguing task, such as a 90-min driving task, can decrease performance on a subsequent Go/NoGo task, as indicated by the observed increased reaction times and omissions.

As described above, it is now well established that mental fatigue negatively impacts both physical and cognitive performances. A number of recent studies have found evidence that acute smartphone use might also negatively affect physical performance, while also bringing about an increase in feelings of mental fatigue^10,13,15^. In this context, the present study aims to investigate the effects of acute smartphone use (i.e., 45 min) on cognitive performance, and in particular on vigilance, attention, and inhibition capacities. We hypothesized that acute use of a smartphone would induce a feeling of mental fatigue associated with an impairment of vigilance, attention, and inhibition.

## Experiment 1

### Methods

#### Participants

Eighty healthy young adults (73 females, 7 males, M_age_ = 20.0, SD = 2.2 years), recruited from the Université de Bourgogne, participated in Experiment 1 and were randomly divided into two groups (i.e., smartphone use or documentary watching). Participants were instructed to get at least six hours of sleep, to refrain from consuming alcohol, and to avoid vigorous physical activity the day before each visit. They were also required not to consume caffeine or nicotine and to avoid using their smartphones for at least three hours before testing. Participants were asked to disclose any medication or acute illnesses, injuries, or infections. These instructions were checked at the beginning of the laboratory visit with a questionnaire. All participants complied with these instructions. Before the experiment, each participant read the information notes and gave their written informed consent. The experiment was conducted in accordance with the most recent version of the Declaration of Helsinki (1964) and was approved by the local Ethics Committee of the Université Bourgogne Franche-Comté (CERUBFC-2021-05-12-010).

#### Experimental procedure

Experiments started between 8:00 a.m. and 11:00 a.m. for all participants and lasted about 1.5 h. Each session took place in a small and quiet room without windows. It started with a short period of familiarization (10 trials) with a PVT, which consisted in responding as fast as possible to the appearance of a visual stimulus (see 2.1.3.3). The participants then completed a questionnaire to evaluate their sleep duration and quality, motivation to perform the experiment, mental fatigue, and drowsiness levels. They then performed the PVT (pretest), followed by either 45 min of smartphone use or a control task (i.e., watching a documentary). Subsequently, after indicating their levels of mental fatigue, drowsiness, boredom, and the perceived workload of using the smartphone or watching the documentary (control task), participants performed the PVT again (posttest). Finally, participants reported the specific activities they were engaged in on their smartphones during the 45-min use period.

#### Experimental tasks

*Smartphone use*. Participants were asked to use their smartphones for 45 min in a way that was as normal as possible in their daily lives. They were free to use their smartphone as they wanted, except for watching videos longer than 3 min and making phone calls. The same room was used for all tasks. The participants were comfortably seated on a chair.

*Control task*. The control task consisted in watching an emotionally neutral documentary on a smartphone. To help prevent the participants from experiencing boredom while watching the documentary, three different documentaries were proposed: “Legacy” by Y. Arthus-Bertrand, “Schumacher” by H.-B. Kammertöns and “Bill Gates” by S. Malterre. Watching movies was recently found to act as a good control intervention to study mental fatigue^34^. The viewing duration was the same as period of smartphone use (i.e., 45 min).

*Psychomotor vigilance task.* The PVT is a simple visual reaction time test developed to evaluate vigilance^35^. Experiment Builder software (SR Research) was used to monitor stimulus presentation. Participants were asked to focus their attention on a red rectangular box in the middle of a black screen. They were instructed to press a button as quickly as possible when a green circle appeared in the center of a red rectangle. This stimulus was displayed for 100 ms, and the reaction time was shown on the screen for 1 s after the button press. If the participant took 500 ms or more to respond, the message "Miss" was displayed on the screen. The stimuli were presented with a random interstimulus interval of between 2 and 10 s. The task consisted of 50 trials and lasted approximately 5 min 20 s. Reaction times faster than 100 ms and those more than two standard deviations above or below the mean were excluded from the analysis.

#### Psychological measures

*Sleep.* The Saint Mary's Hospital Sleep questionnaire was administered to assess the participants' sleep quality and duration the night before each experimental session. This questionnaire comprises 14 items that delve into various aspects of sleep quality, including depth, nighttime awakenings, satisfaction, morning alertness, difficulty falling asleep, and early awakenings.

*Motivation.* Motivation is defined as “the attribute that moves us to do or not to do something”^36^. Motivation to complete the experiment was measured using a motivation scale developed by Matthews et al.^37^. The questionnaire consisted of seven questions related to intrinsic motivation (e.g., "I want to do my best") and seven related to extrinsic motivation (e.g., "I only did this task for an external reward"). Participants could choose among five possible responses to each question (0 = not at all, 1 = a little bit, 2 = somewhat, 3 = very much, 4 = extremely). The scores for each motivation type ranged from 0 to 28.

*Subjective workload.* The National Aeronautics and Space Administration Task Load Index (NASA-TLX) was used to evaluate subjective workload^38^. The NASA-TLX consists of six subscales: mental demand (how much mental and perceptual activity was required?), physical demand (how much physical activity was required?), temporal demand (how much time pressure did you feel due to the rate or pace at which the task occurred?), performance (how much successful do you think you were in accomplishing the goals of the task set by the experimenter?), effort (how hard did you have to work to achieve your level of performance?), and frustration (how irritating or annoying did you find the task?). Participants rated each item on a scale divided into 20 equal intervals anchored by a bipolar descriptor (e.g., high/low). The scores were multiplied by 5, resulting in a final score of between 0 and 100 for each subscale.

*Mental fatigue level.* A visual analog scale (VAS) was used to measure feelings of mental fatigue before and after smartphone use and before and after documentary watching. The VAS consisted of a 100-mm line with bipolar end anchors (0 mm = "Not tired at all"; 100 mm = "Extremely tired"). Participants were asked the question: "How mentally fatigued do you feel right now?" and were told to place a mark on the line to indicate their current level of fatigue. The VAS score was determined by measuring the distance between the first anchor (0 mm: "Not tired at all") and the mark placed by the participant.

*Drowsiness level.* A VAS was used to measure feelings of drowsiness before and after smartphone use and before and after documentary watching. This VAS consisted of a 100-mm line with bipolar end anchors (0 mm = "Not drowsy at all"; 100 mm = "Extremely drowsy"). Participants were asked the question: "How drowsy do you feel right now?" and were told to place a mark on the line to indicate their current level of drowsiness. The VAS score was determined by measuring the distance between the first anchor (0 mm: "Not drowsy at all") and the mark placed by the participant.

*Boredom.* Feelings of boredom after smartphone use and documentary watching were evaluated by using a VAS with bipolar end anchors (0 mm = "Not bored at all"; 100 mm = "Extremely bored"). Participants were asked the question: "How bored do you feel right now?" and were instructed to place a mark on the line to indicate their current level of boredom. The VAS score was determined by measuring the distance between the first anchor (0 mm: "Not bored at all") and the mark placed by the participant.

*Activities performed on smartphone.* Participants reported on a questionnaire the specific activities they were engaged in on their smartphone during the 45-min use period. Subsequently, we categorized these activities into four different groups: "Social network," "Internet search," "Productivity" (which included activities such as email, diary management, and writing), and "Games".

#### Statistics

The data are presented as means ± standard errors of the mean. When sphericity was violated, the degrees of freedom were adjusted using the Greenhouse–Geisser method (the corrected degree of freedom and *p*-values are reported). Only significant results are reported unless the absence of significance is relevant to the hypotheses tested.

*T* tests were used to evaluate differences in sleep duration, motivation, boredom, and NASA-TLX scores participants in the control versus smartphone groups.

Effects on the mental fatigue VAS, drowsiness VAS, and performances during the PVT (reaction times, anticipation, omissions, and global errors [omissions + anticipations]) were evaluated using a two-way mixed-model repeated-measures 2 × 2 ANOVA with group (Control, Smartphone) as between-subject factor, and time (Pre, Post) as within-subject factor.

All analyses were performed using JASP (Version 0.17.1.0) [Windows software]. Significant interactions were further analyzed by means of contrast tests with Bonferroni correction, and adjusted *p*-values were reported. Partial eta squared was calculated for each repeated-measures ANOVA. Thresholds for small, moderate, and large effects were set at 0.01, 0.07, and 0.14, respectively^39^. Cohen’s *d* was calculated for each *t* test. Thresholds for small, moderate, and large effects were set at 0.2, 0.5, and 0.8, respectively^39^.

## Results

### Psychological measures

*Sleep duration.* No difference in sleep duration was observed, *t*(78) = − 0.604, *p* = 0.547, *d* =  − 0.135, between participants in the smartphone condition and those in the documentary condition (505 min ± 88 *vs*. 495 min ± 62, respectively).

*Motivation.* There was no difference in intrinsic, *t*(78) =  − 0.801, *p* = 0.426, *d* =  − 0.179 (17.5 ± 0.5 vs. 18.0 ± 0.5), or in extrinsic motivation, *t*(78) = – 1.340, *p* = 0.184, *d* =  − 0.300 (17.7 ± 0.4 *vs*. 18.6 ± 0.5) to perform the session between the two experimental conditions.

**Figure 1:**
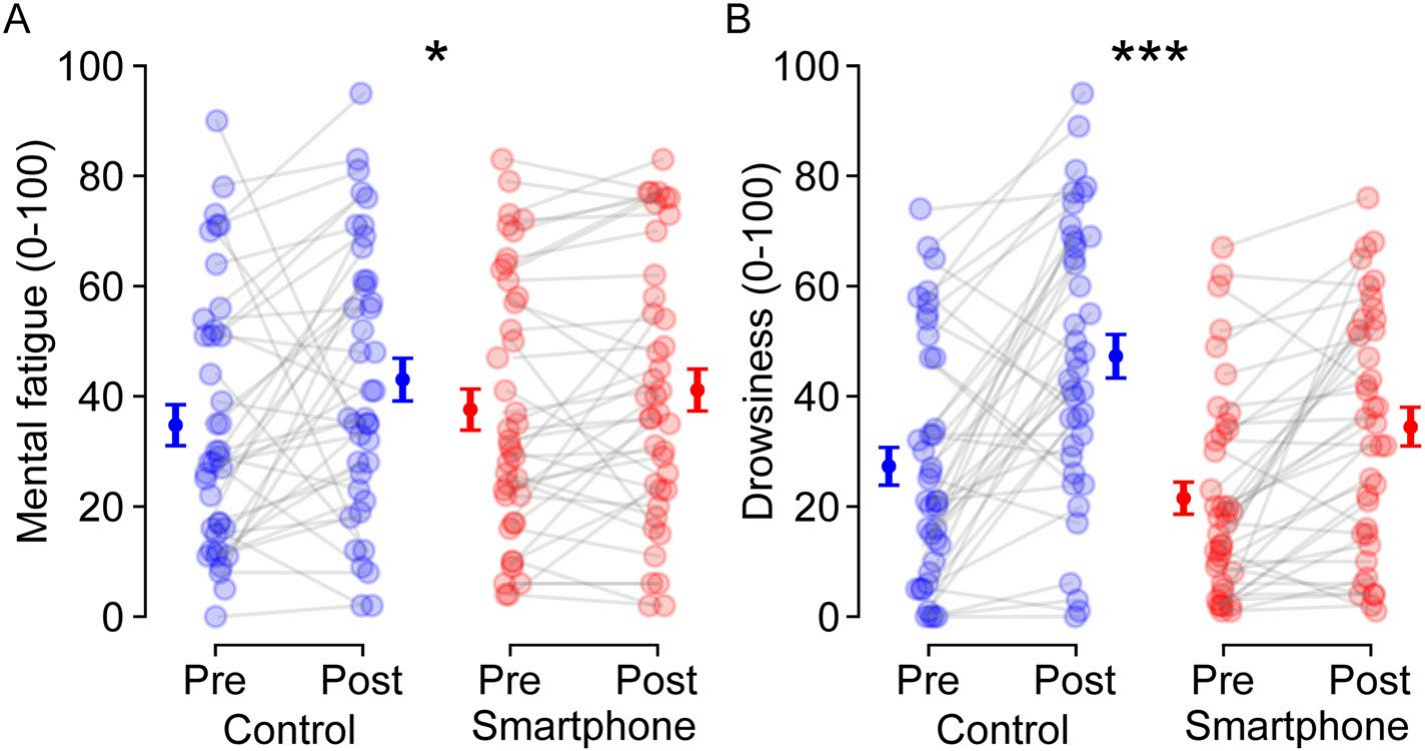
Effects of smartphone use on subjective mental fatigue (**A**) and drowsiness (**B**) evaluated using a visual analog scale. Individual (*N* = 40) data are represented with empty markers and means ± SEM as filled markers. * and *** indicate main effects of time significant at *p* < 0.05 and p < 0.001, respectively.

*Mental fatigue.* Mental fatigue increased following both smartphone use and documentary watching [time effect: *F*(1, 78) = 9.809, *p* = 0.011, ${\eta }_{p}^{2}$ = 0.080; from 36.2 ± 2.6 to 42.1 ± 2.7]. However, neither a group effect, *F*(1, 78) = 0.009, *p* = 0.924, ${\eta }_{p}^{2}$ < 0.001, nor a group × time interaction, *F*(1, 78) = 1.087, *p* = 0.300, ${\eta }_{p}^{2}$ = 0.014, were observed (Fig. 1a).

*Drowsiness.* Drowsiness increased following both smartphone use and documentary watching [time effect: *F*(1, 78) = 50.505, *p* < 0.001, ${\eta }_{p}^{2}$ =  − 0.393; from 24.4 ± 2.3 to 40.8 ± 2.7], and the participants in the control group were more drowsy than those in the smartphone group [group effect: *F*(1, 78) = 50.505, *p* < 0.001, ${\eta }_{p}^{2}$ =  − 0.393; Control: 37.3 ± 3.2, Smartphone: 28.0 ± 2.9]. However, no group × time interaction was observed, *F*(1, 78) = 2.352, *p* = 0.129, ${\eta }_{p}^{2}$ = 0.029 (Fig. 1b).

*Boredom.* Analyses performed on boredom did not reveal any differences, *t*(78) =  − 0.960, *p* = 0.340, *d* =  − 0.214, between participants’ feeling of boredom after smartphone use and documentary watching (38.1 ± 3.0 *vs*. 33.7 ± 3.1, respectively).

*Perceived workload.* Analyses performed on the NASA-TLX revealed that smartphone use was perceived as less temporally demanding than documentary watching, *t*(78) = 3.151,* p* = 0.002, *d* = 0.705. In addition, participants perceived their performance as being higher for smartphone use than for documentary watching, *t*(78) = – 3.066,* p* = 0.003, *d* =  − 0.686 (Table 1).

**Table 1:** The perceived workload for the Control and Smartphone groups (Experiment 1).

	Mental demand	Physical demand	Temporal demand	Frustration	Performance	Effort
Control	20.4 (± 1.9)	10.1 (± 1.6)	45.6 ** (± 4.1)	21.0 (± 3.2)	82.4 ** (± 2.3)	17.0 (± 1.8)
Smartphone	16.0 (± 2.4)	7.1 (± 1.3)	27.5 (± 4.0)	20.3 (± 4.1)	91.8 (± 2.5)	13.8 (± 2.5)

**: Significant main effect of group (*p* < 0.01). Data are presented as means (± SEM).

*Activities performed on smartphone.* Participants reported spending 32.9 min (± 1.9) on social network, 5.7 min (± 1.5) on Internet search, 1.6 min (± 0.6) on productivity applications, and 4.6 min (± 1.8) on games.

#### Behavioral performances for vigilance

**Figure 2:**
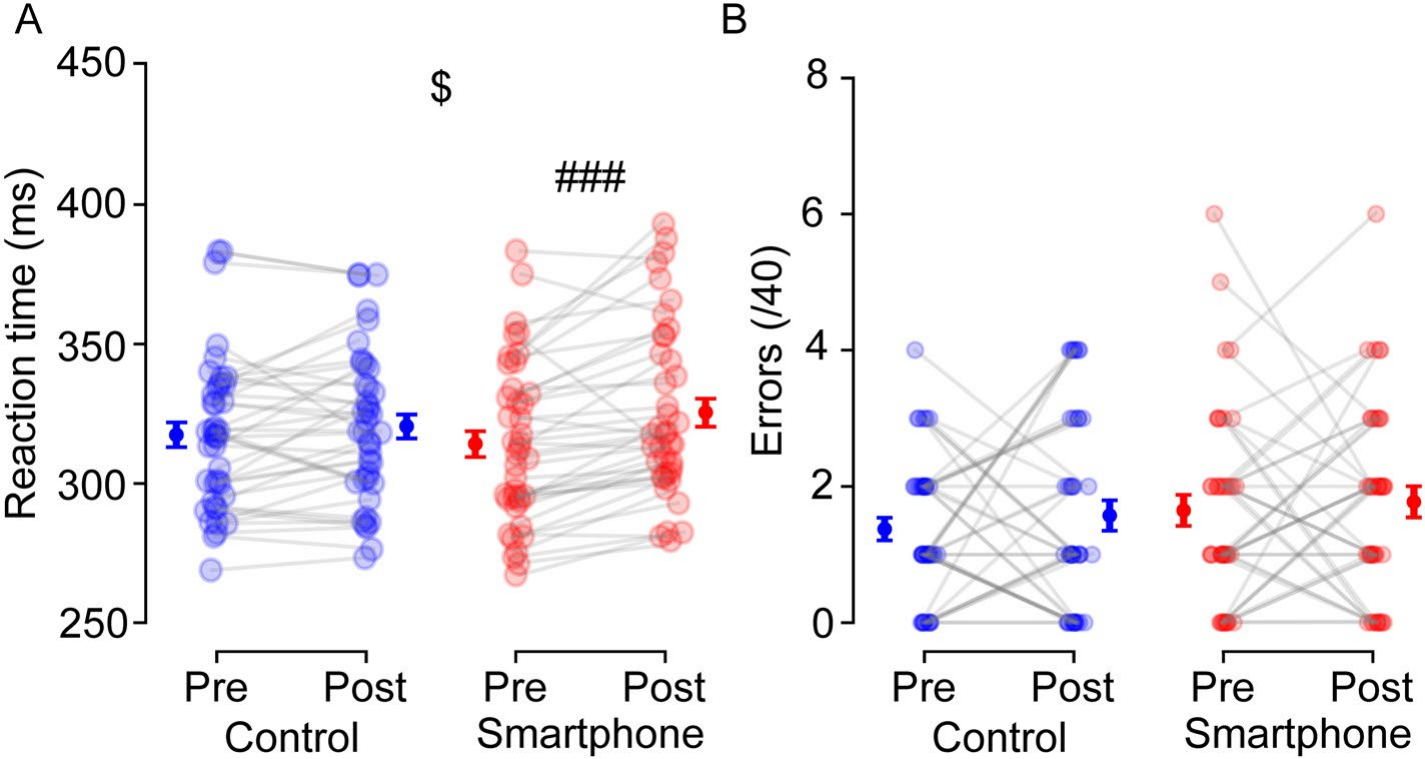
Effects of smartphone use on reaction time (**A**) and global errors (**B**) during the psychomotor vigilance task. Individual (*N* = 40) data are represented with empty markers and means ± SEM as filled markers. $: Significant group × time interaction. ###: Difference between pre and post within the same group (*p* < 0.001).

The analyses run on reaction times revealed a significant group × time interaction, *F*(1, 78) = 6.190,* p* = 0.015, ${\eta }_{p}^{2}$ = 0.076, indicating a significant increase in reaction times after smartphone use, *t*(39) = – 4.646,* p* < 0.001, *d* =  − 0.735 (314.1 ms ± 4.6 to 325.5 ms ± 5.0), but not after documentary watching, *t*(39) = – 1.400,* p* = 0.169, *d* =  − 0.221 (317.37 ms ± 4.3 to 320.5 ms ± 4.3, Fig. 2a). However, there were no significant effects (all *ps* > 0.17, ${\eta }_{p}^{2}$ < 0.03) on omissions, anticipations, or global errors (missed targets + anticipations, Fig. 2b).

## Experiment 2

### Material and methods

#### Participants

Eighty healthy young adults (73 females, 7 males, M_age_ = 19.5, SD = 2.8 years), recruited from the Université de Bourgogne and different from those who performed Experiment 1, participated in Experiment 2. They were randomly divided into two groups (i.e., smartphone use and documentary watching). All information regarding inclusion–exclusion criteria, prelaboratory visits, and ethics is identical to Experiment 1.

#### Experimental procedure

The experimental procedure was similar to that used in Experiment 1, except that the PVT was replaced by a Go/NoGo task, which evaluates inhibition capacities (see Sect. “Experimental procedure”).

The session began with a brief habituation (30 trials) to the Go/NoGo task (see 2.1.3.3). Participants completed a questionnaire to evaluate their sleep duration and quality, motivation, levels of mental fatigue, and drowsiness. Next, they performed the Go/NoGo task (pretest), followed either by a 45-min period of smartphone use or a control task of the same duration (watching a documentary). Participants reported their levels of mental fatigue, drowsiness, boredom, and the subjective workload induced by either smartphone use or the control task. Then, participants performed the Go/NoGo task again (posttest). Finally, participants reported the specific activities they were engaged in on their smartphone during the 45-min use period.

#### Experimental tasks

Smartphone use and control task. These tasks were the same as in Experiment 1 (see Sect. “Experimental tasks”).

*Go/NoGo task.* The Go/NoGo task is a cognitive task currently used to evaluate inhibition capacities. Experiment Builder software (SR Research) was used to monitor stimulus presentation. A white fixation cross was displayed continuously at the center of a black screen. The stimulus was either a white triangle (Go stimulus) or a white circle (NoGo stimulus) and was presented on the left or right side of the fixation cross. The stimuli were displayed for 100 ms, with an interstimulus interval of 2500 ms. The task consisted of 150 trials and lasted approximately 6 min 30. There were 120 (80%) Go stimuli and 30 (20%) NoGo stimuli. When a Go stimulus was displayed, the participants had to respond by pressing the space bar with their right index finger, whereas they were told not to react to NoGo Stimuli. The left or right positions were used equally often and in random order. Participants were instructed to respond as quickly and as accurately as possible. Reaction times faster than 100 ms or two or more standard deviations above or below the mean were excluded from the analysis. Errors on the Go and NoGo trials were analyzed separately.

#### Psychological measures

The psychological measures were the same as in Experiment 1 (see Sect.  “Psychological measures”).

#### Statistics

The statistical analyses performed to assess differences in psychological measures were the same as in Experiment 1. To analyze the Go/NoGo performance (reaction times for Go trials, errors for Go trials, and errors for NoGo trials), we used two-way mixed-model repeated-measures 2 × 2 ANOVAs with group (Control, Smartphone) as a between-subject factor, and time (Pre, Post) as a within-subject factor were used.

### Results

#### Psychological measures

*Sleep duration.* No difference in sleep duration, *t*(78) = 0.441, *p* = 0.661, *d* =  − 0.099, was observed between participants in the smartphone condition and those in the documentary condition (484 min ± 9.4 vs. 490 min ± 9.8, respectively).

*Motivation.* There were no differences in either intrinsic, *t*(78) = 0.881, *p* = 0.381, *d* = – 0197 (18.1 ± 0.5 vs. 17.5 ± 0.4) or in extrinsic motivation, *t*(78) =  − 0.557, *p* = 0.579, *d* =  − 0.125 to perform as a function, smartphone vs documentary (18.2 ± 0.4 vs. 18.5 ± 0.4, respectively).

**Figure 3:**
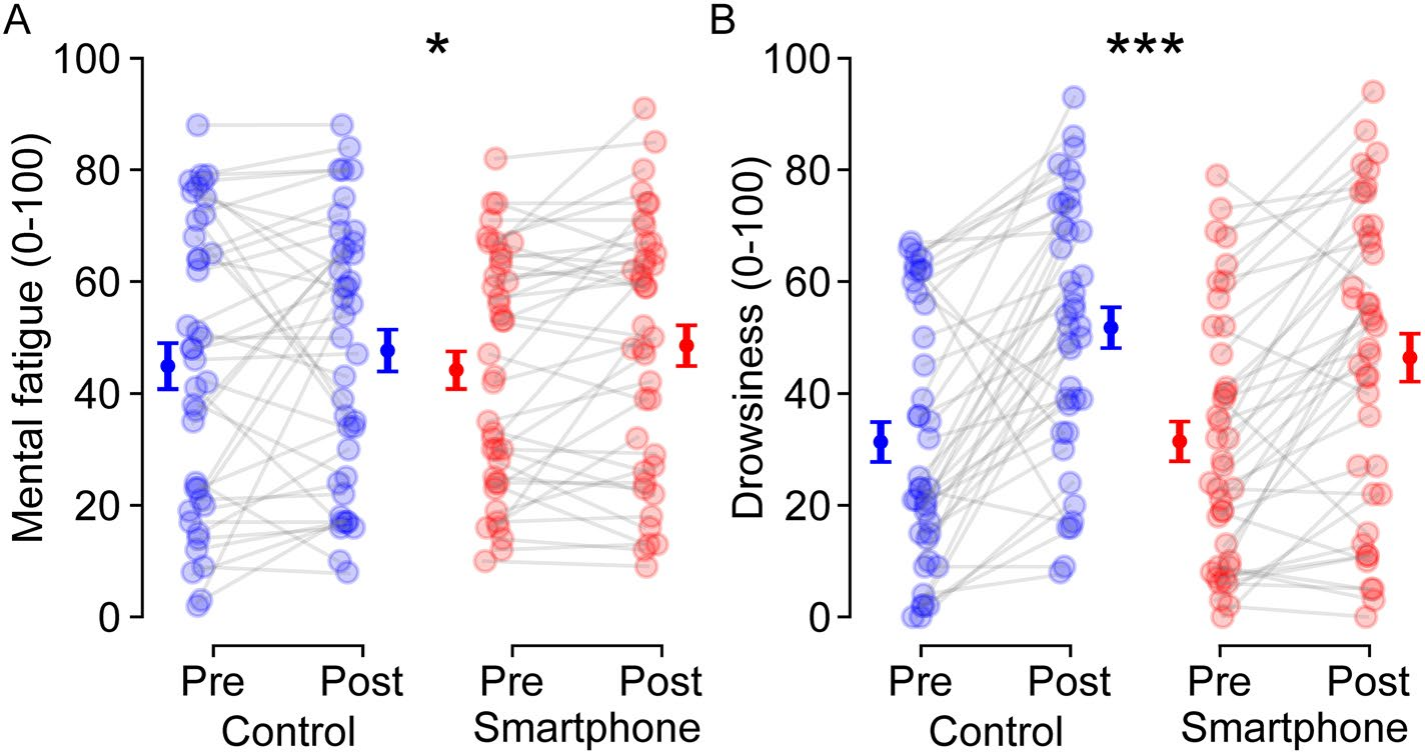
Effects of smartphone use on subjective mental fatigue (**A**) and drowsiness (**B**) evaluated using a visual analog scale. Individual (*N* = 40) data are represented with empty markers and means ± SEM as filled markers. * and *** indicate main effects of time significant at *p* < 0.05 and *p* < 0.001, respectively.

*Mental fatigue.* Mental fatigue increased following both smartphone use and documentary watching [time effect: *F*(1, 78) = 3.978, *p* < 0.050,${\eta }_{p}^{2}$ = 0.049; from 44.5 ± 2.6 to 48.1 ± 2.6]. However, neither a group effect, *F*(1, 78) < 0.001, *p* = 0.988, ${\eta }_{p}^{2}$ < 0.001, nor a group × time interaction, *F*(1, 78) = 0.199, *p* = 0.657, ${\eta }_{p}^{2}$ = 0.002, were observed (Fig. 3a).

*Drowsiness.* Drowsiness increased following both smartphone use and documentary watching [time effect: *F*(1, 78) = 60.665, *p* < 0.001, ${\eta }_{p}^{2}$ = 0.438; from 31.4 ± 2.5 to 49.0 ± 2.8]. However, neither a group effect, *F*(1, 78) = 0.296, *p* = 0.588, ${\eta }_{p}^{2}$ = 0.004, nor a group × time interaction, *F*(1, 78) = 1.457, *p* = 0.231, ${\eta }_{p}^{2}$ = 0.018, were observed (Fig. 3b).

*Boredom.* Analyses performed on boredom did not reveal any differences, *t*(78) =  − 0.691, *p* = 0.492, *d* =  − 0.154, in the results after smartphone use and documentary watching (39.3 ± 3.6 vs. 36.1 ± 3.0, respectively).

*Subjective workload.* Analyses performed on the NASA-TLX revealed that smartphone use was perceived as less temporally demanding than documentary watching, *t*(78) = 2.430, *p* = 0.002, *d* = 0.543. In addition, participants perceived their performance as being higher for smartphone use than for documentary watching, *t*(78) = – 3.125, *p* = 0.003, *d* =  − 0.699 (Table 2).

**Table 2:** The perceived workload for the Control and Smartphone use groups (Experiment 2).

	Mental demand	Physical demand	Temporal demand	Frustration	Performance	Effort
Control	28.5 (± 3.3)	12.4 (± 2.4)	46.6 ** (± 3.5)	25.1 (± 3.8)	81.1 ** (± 2.4)	24.4 (± 3.5)
Smartphone	21.0 (± 3.0)	10.0 (± 1.7)	34.3 (± 3.7)	18.0 (± 3.1)	91.6 (± 1.9)	19.3 (± 3.1)

**: Main effect of group (*p* < 0.01). Data are presented as means (± SEM).

*Activities performed on smartphone.* Participants reported spending 32.2 min (± 1.9) on social network, 6.7 min (± 1.6) on Internet search, 1.5 min (± 0.6) on productivity applications, and 4.6 min (± 1.5) on games.

#### Behavioral performances for inhibition capacities

**Figure 4:**
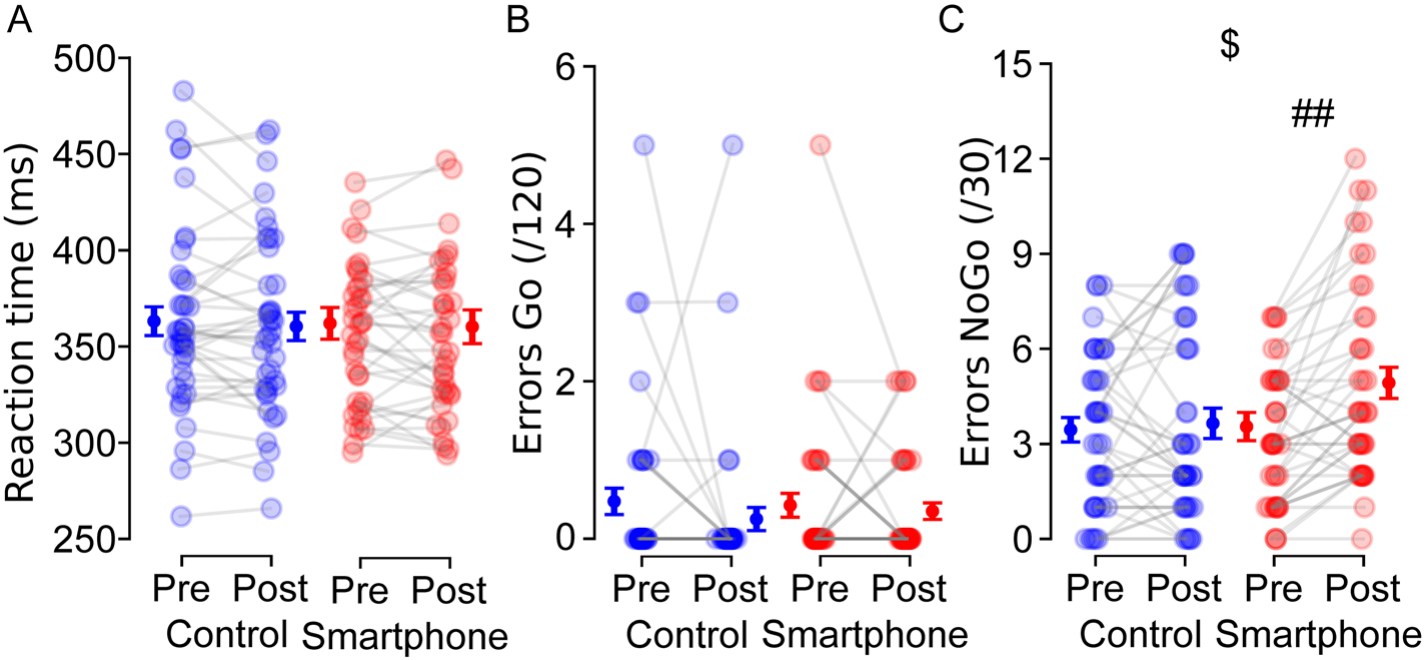
Effects of smartphone use on reaction times (**A**), errors for Go trials (**B**), and NoGo trials, (**C**) during the Go/NoGo task. Individual (N = 40) data are represented with empty markers and means ± SEM as filled markers. $: Significant group × time interaction. ##: Difference between pre and post within the same group (*p* < 0.01).

For reaction times, neither a significant time effect, *F*(1, 78) = 1.098,* p* = 0.298, ${\eta }_{p}^{2}$ = 0.014, nor a group × time interaction, *F*(1, 78) = 0.048,* p* = 0.828, ${\eta }_{p}^{2}$ = 0.001, were observed (Fig. 4a). While there were no significant effects on errors for Go trials (all *p*s > 0.20, ${\eta }_{p}^{2}$ < 0.021, Fig. 4b), the analyses on NoGo trials revealed a group × time interaction, *F*(1, 78) = 5.231,* p* = 0.025, ${\eta }_{p}^{2}$ = 0.063, thus indicating an increase in errors after smartphone use, *t*(78) = –3.075, *p* = 0.008, *d* =  − 0.486 (3.55 ± 0.44 *vs*. 4.92 ± 0.49) but not after documentary watching, *t*(78) =  − 0.312, *p* = 1.000, *d* =  − 0.049 (3.45 ± 0.38 *vs*. 3.65 ± 0.48, Fig. 4c).

## Discussion

The present study aimed to examine the impact of acute smartphone use on vigilance, attention, and inhibition capacities. Participants reported an increase in subjective mental fatigue, as assessed by a VAS, following smartphone use. However, it is noteworthy that a similar increase in mental fatigue was also observed in a control group whose participants watched a documentary. After 45 min of smartphone use, the results indicated a decline in vigilance, as evidenced by increased reaction times during the PVT. Interestingly, reaction times and the number of omissions remained constant during the Go/NoGo task, suggesting that attention abilities were preserved. However, an increase in errors during the NoGo trials indicated impaired inhibition capacities. This study highlights the negative impact of acute smartphone use on vigilance and inhibition capacities. While feelings of mental fatigue increased in both groups, performance declined only after acute smartphone use. This objectively indicates that mental fatigue occurred only after acute smartphone use. As the increased feeling of mental fatigue occurred in both conditions, even when there were no detrimental effects on cognitive performance, future studies should attempt to identify the electrophysiological markers of the presence of mental fatigue after acute smartphone use.

## Evaluation of mental fatigue

In line with other recent studies, our research has shown that using smartphones can increase feelings of mental fatigue^10,13^. However, we observed a similar increase in feelings of mental fatigue in the control group. In addition, we observed that both smartphone use and documentary watching led to increased drowsiness. Drowsiness is the intermediate state between awareness and sleep^40^. Since all participants reported having more than six hours of sleep the night before the experiment and the experimental tasks were relatively short (45 min), it is unlikely that drowsiness was related to sleep deprivation. One possible explanation for the drowsiness experienced by the participants relates to the experimental conditions themselves. The study was conducted in a windowless room with a relatively low lighting level and this may have contributed to the onset of drowsiness. Although mental fatigue and drowsiness are two distinct and dissociable phenomena^41^, they can manifest themselves in similar ways, such as feeling tired and experiencing reduced alertness. Even if subjective measurements like VAS have been widely adopted for assessing mental fatigue^27^, it is necessary to back them up with objective measures, such as behavioral performances, or physiological measurements such as electroencephalography and/or heart rate variability, in order to confirm the link between acute smartphone use and mental fatigue.

## Effects of acute smartphone use on cognitive performances

The effects of smartphone use have mainly been studied in the context of chronic use, and many different effects have been reported. In addition to the appearance of sleep or emotional disorders, chronic smartphone use can lead to impaired cognitive performance. The present study aimed to evaluate the effects of acute smartphone use on attentional and inhibition capacities. We observed (i) an increase in reaction times during the PVT unaccompanied by any changes in error rate after acute smartphone use, thus indicating impaired vigilance, and (ii) an increase in NoGo errors in the Go/NoGo task showing an impairment in inhibition capacities. Such differences were not observed in the control group.

One previous study found an increase in reaction times and errors during a Stroop task after 30 and 45 min of acute smartphone use^15^. In contrast, another study reported an increase in reaction times without any increase in errors^12^. An increase in reaction times suggests slower processing and delayed responses during the Stroop task. The authors attributed the performance impairment, which was also observed for physical tasks, to the presence of mental fatigue as evaluated with a VAS. The performance deterioration might have been due to a decrease in attentional resources and difficulties in inhibiting the automatic response when reading the word. The increased reaction times might have been attributable to reduced attentional resources, especially in selective attention, and difficulties in inhibiting the automatic reading response^42^. Moreover, the increased errors during the Stroop task suggest a reduced ability to inhibit automatic responses and overcome interference. In the present study, the increase in errors on the NoGo trials during the Go/NoGo task confirms the impairment of inhibition capacities after acute smartphone use. The maintenance of reaction times and errors for Go trials indicates that participants continued to try to perform the task correctly but found it more difficult to inhibit a motor response when presented with an irrelevant stimulus.

At the same time, the increase in reaction times during the PVT after 45 min of smartphone use, without this having any effect on errors, testified to impaired vigilance. This impairment has already been observed in the presence of mental fatigue. Smith et al.^27^ not only found an increase in reaction times overtime during a prolonged PVT (i.e., 45 min) but also an increase in reaction times, unaccompanied by an effect on errors, during a PVT after a mentally fatiguing task of 45 min (e.g., Stroop task).

## Potential role of the anterior cingulate cortex in performance impairment

Studies on mental fatigue revealed changes in cortical activity, in particular modulations of activity in the prefrontal cortex. Among the structures concerned, there is a decrease in the activity of the anterior cingulate cortex (ACC), a brain region involved in attentional control and conflict resolution^43,44^. It has been found that a decrease in ACC activity was linked to an increase in reaction times during a PVT^45^. The observed detrimental impact of acute smartphone use on reaction times during the PVT may have been due to a reduction in ACC activity, potentially associated with the presence of mental fatigue. Although this hypothesis could not be validated in the present study, some neuroimaging studies have shown a decrease in ACC activity in intensive smartphone users^46,47^. Based on the similarity between the effects usually observed and those observed with acute smartphone use, and also considering the imaging data, there is reason to hypothesize that the use of smartphones can affect the activity of the ACC and lead to impaired performance. The ACC is also highly activated during response inhibition^43^. Lesions or damage to the ACC can lead to deficits in inhibitory control and difficulties in suppressing inappropriate responses^48^. However, further neuroimaging studies (functional magnetic resonance imaging or near-infrared spectroscopy) are needed in order to confirm that smartphone use leads to a decrease in ACC activity that may be responsible for the impairment of attentional and inhibition capacities.

## Neuronal substrates of performance impairment: exploring the involvement of the prefrontal cortex

The prefrontal cortex, including the ACC, is considered to be a key brain area involved in the phenomenon of mental fatigue^49^. Many different studies have reported an association between prefrontal activation and fatigue following prolonged, demanding physical or mental exertion. A recent study also revealed that the induction of mental fatigue due to prolonged cognitive work is associated with an increase in glutamate accumulation in the lateral prefrontal cortex^50^. Regarding the diminished activation of the dorsolateral prefrontal cortex (DLPFC), Lim et al.^45^ found that, in addition to a decrease in ACC activity, mental fatigue might induce a reduction in the activity of the middle frontal gyrus, a part of the DLPFC. Consequently, it has been hypothesized that a functional adaptation takes place due to the costs associated with the effort made. The fatigue-induced decline in motor performance is regulated by means of the crucial role played by inhibitory projections from the DLPFC to the motor cortex. It is worth mentioning that the application of transcranial direct current stimulation (tDCS) to the left DLPFC has been shown to mitigate the adverse impacts of mental fatigue on athletic performance^51^. Moreover, emerging research suggests that intensive smartphone users exhibit reduced DLPFC activity^9,52^, associated with an impairment in inhibition processing^9^. To the best of our knowledge, no study has as yet investigated the changes in brain activity related to acute smartphone use. However, based on the literature, a decrease in DLPFC activity after acute smartphone use would potentially be a way of accounting for the impairment in the ability to inhibit a motor response during nonrelevant trials in a Go/NoGo task and the increase in errors during NoGo trials. Regarding the accumulation of glutamate in the lateral prefrontal cortex, Wiehler et al.^50^ reported a higher glutamate concentration in this brain area following daylong cognitive work. This glutamate accumulation has been correlated with attention deficit in hyperactivity disorder^53^ and impulsivity in healthy adults^54^, and is likely linked to the deterioration in vigilance and inhibition capacity observed in our study.

## Limitations and perspectives

While behavioral performance declined only after acute smartphone use, the feeling of fatigue increased both with acute smartphone use and when watching a documentary. Furthermore, drowsiness increased in both groups. We cannot exclude the possibility that the increased drowsiness following acute smartphone use and watching a documentary could be a confounding factor in the rating of mental fatigue. Our findings suggest that using only subjective evaluations of tiredness and lack of energy may not be the best way of monitoring the progression of mental fatigue. It would therefore be worst investigating the effects of mental fatigue on cognitive and physical performance, not just by assessing subjective fatigue indicators (i.e., feelings of mental fatigue) but also by examining objective manifestations of fatigue. These objective manifestations of fatigue could be captured through changes in performance or neurophysiological variables. Additionally, future studies should consider monitoring the educational and socioeconomic levels of the population under investigation to better characterize the observed effects. Understanding how these factors may interact with smartphone use and other cognitive tasks can provide a more comprehensive picture of the impact of mental fatigue. Moreover, while we evaluated sleep, we did not evaluate for the presence of emotional disorders or other mental health disorders in our participants, which can also considerably affect cognitive performance^55^. In future investigations, it might be of value to consider controlling for these parameters. Furthermore, we did not control for participants' typical average duration of smartphone use. It is perfectly conceivable that individuals accustomed to prolonged smartphone use may respond differently to acute smartphone use.

## Conclusion

The findings of this study demonstrate that acute smartphone use (i.e., 45 min) induces an increase in the sensation of mental fatigue and drowsiness. However, the increase in the feeling of mental fatigue and drowsiness was comparable in magnitude after viewing a documentary (i.e., control group). Behavioral results indicated a decline in vigilance and inhibition capacities after acute smartphone use only. Further studies are needed to (i) confirm the presence of mental fatigue after acute smartphone use based on the use of objective measures (e.g., electroencephalography, heart rate variability) and (ii) determine the neurophysiological mechanisms underlying the negative effects of smartphone use on attention and inhibition processes. Finally, individuals may need to be more aware of the impact of smartphone use on their cognitive abilities and take appropriate measures to mitigate any negative effects.

## Data Availability

The datasets generated and analyzed during the current study are available from the corresponding author upon reasonable request.
